# Proinflammatory Markers in Prediction of Posttraumatic Psychological Symptoms: A Prospective Cohort Study

**DOI:** 10.1155/2008/640659

**Published:** 2008-12-22

**Authors:** Alasdair George Sutherland, Gary A. Cameron, David A. Alexander, James D. Hutchison

**Affiliations:** ^1^Department of Orthopaedics, School of Medicine and Dentistry, University of Aberdeen, Polwarth Building, Foresterhill, Aberdeen AB25 2ZD, Scotland, UK; ^2^Department of Medicine and Therapeutics, University of Aberdeen, Polwarth Building, Foresterhill, Aberdeen AB25 2ZD, Scotland, UK; ^3^Aberdeen Centre for Trauma Research (ACTR), The Robert Gordon University, Schoolhill, Aberdeen AB10 1FR, Scotland, UK

## Abstract

*Introduction*. Posttraumatic psychopathology (PTP) describes the spectrum of conditions that can complicate the recovery from commonly occurring musculoskeletal trauma. There is a clear association with the activation of the hypothalamic-pituitary-adrenal axis (HPAA), and we wished to examine the predictive value of proinflammatory markers of the HPAA and of the GABA, which acts as an inhibitory regulator. *Methods*. Levels of proinflammatory markers and GABA were measured in 84 patients who had suffered musculoskeletal injuries requiring hospitalisation. PTP was assessed by the use of the General Health Questionnaire (GHQ) at presentation and again at two- and six-month reviews. *Results*. Significant psychological disturbance was noted in 39% of patients at two months and falling back to 18% by six months. There was no correlation between any of the markers tested at presentation and PTP at follow-up. *Discussion*. The HPAA response to trauma and the development of PTP are extremely complex. It is unlikely that a simple blood assay will provide significant predictive information, while incident specific information and patient perception are of more practical use.

## 1. INTRODUCTION

 Psychological disturbance after traumatic injury is common, and it is now well recognised that disorders such as posttraumatic stress disorder (PTSD) are not confined to combat veterans or the survivors of major civil accidents [1–3]. There is a spectrum of posttraumatic problems; from short-lived adjustment conditions such as acute stress disorder (ASD) to full PTSD, and a range of comorbidities such as anxiety and depression can coexist with specific posttraumatic symptoms. Rather than focusing upon only the most severe extreme, we prefer to consider the full spectrum of posttraumatic psychological (PTP) symptoms to describe the range of problems that may occur after a traumatic event [2]. We have previously demonstrated that 2 months after a musculoskeletal injury up to 46% of patients have demonstrable psychopathology, falling to 22% by six months [4]. This PTP is important to the treating trauma surgeon, as there is a clear relationship between psychopathology and impaired functional recovery. Although it is not clear that primary treatment of the psychological problems will necessarily yield an improvement in recovery from the physical aspects of the injury [5, 6], it is certainly clear that holistic care of the injured patient must include management of their psychological state. The ability to predict which patients are at highest risk of PTP, including PTSD, would allow scarce resources to be targeted at those most in need.

 Previous work from our and other units has characterised aspects of the injury, including injury severity and the patient's experience of the accident, which are associated with increased risk of PTP [4, 7–9]. The awareness of the centrality of the hypothalamic-pituitary-adrenal axis (HPAA) to the development of PTP [10] has led to a search for biological markers that might predict a tendency to develop PTP that is independent of the injury itself. A recent paper has suggested that low levels of *γ*-amino butyric acid (GABA) [11], an inhibitory modulator of the HPAA, may predict PTSD after road traffic accidents. The proinflammatory cytokines interleukin-6 and its soluble receptor (IL-6 and sIL-6r) and tumour necrosis factor-*α* (TNF-*α*) have been identified as indicators of the inflammatory response to physical stress and HPA disruption [12–17]. There is some contradictory evidence for elevation of these cytokines in patients with established PTSD [18–20] and posttraumatic psychopathology [21]. The relationship between psychiatric illness and cytokine disturbance has been more widely studied, particularly with regard to schizophrenia [22, 23], but it remains to be seen how this can be integrated in the understanding of changes in PTP. GABA has an important inhibitory role in the function of the HPAA [24], and low levels have been associated with increased risk of development of acute PTSD [11, 25].

We wished to study the relationship between GABA and a range of proinflammatory markers, which also reflect HPAA function, and the development of PTP in our centre.

## 2. MATERIALS AND METHODS

 As part of a wider study of trauma outcomes, a cohort of patients treated in the orthopaedic trauma unit of Aberdeen Royal Infirmary were approached for recruitment. Patients eligible for entry were aged 17–70, with at least one musculoskeletal injury. Head injuries are known to affect psychological perception of injuries and so those with significant head injuries (unconscious more than 15 minutes, Glasgow Coma Scale less than 13) were excluded. Patients with fragility fractures were also excluded. Patients were recruited within 48 hours of their injury, by means of discussion, information sheet, and informed consent record. They were asked to complete the General health Questionnaire (GHQ) at initial presentation, and gave an early morning sample of blood. Demographic data related to their injuries and backgrounds were collected, including injury severity score (ISS) [26], the new injury severity score (NISS) [27, 28], the revised trauma score (RTS) [29], and the TRISS methodology score [30]. The study had the full approval of the local research ethics committee.

The GHQ is a validated and robust self-administered screening measure to detect psychiatric disorders in community and nonpsychiatric clinical settings [31, 32]. It is a 28-item questionnaire, producing a total score (a higher score representing more severe psychiatric disturbance). The total score further allows a threshold to be applied that defines “caseness”—the situation where it is likely that clinical examination by a mental health specialist would identify a genuine psychiatric condition. This does not define that the patient has a specific psychiatric diagnosis (this would require a two-stage approach), but it is a useful tool for identifying patients who would be so defined. The threshold for this caseness definition is generally taken as a score of 5 and above (out of 28) [31]. In a posttraumatic setting, there is often extensive symptom overlap, and the GHQ allows us to identify those patients with a psychiatric disturbance without narrowing the focus to one or more specific diagnoses (e.g., PTSD).

 Blood was drawn between 0730 and 0830 for each patient, in order to reduce the potential confounding effect of circadian rhythmns in production of biomarkers under investigation. Sampling used a tourniquet and vacutainer technique, taking 20 mL in two-clotting vacutainers. The clotted samples were centrifuged at 2900 rpm for 15 minutes, and the serum carefully transferred to nonsterile 5 mL vials (eppendorfs) using a microtitre pipette (Gilson). The serum samples were stored at −40°C for no more than 24 hours before being transferred to a −70°C freezer until required, when they were allowed to thaw at room temperature. TNF-*α*, IL-6, and sIL-6r assays were carried out using solid-phase enzyme-linked imunosorbant Assay assay (ELISA) kits (Quantikine, R&D Systems Inc, Minneapolis, Minn, USA, distributed in the UK by R&D Systems Europe, Abingdon, Oxon, UK). CRP assays were carried out in the routine microbiology laboratories of Aberdeen Royal Infirmary. Plasma GABA levels were determined using an LC-MS-MS chromatography method.

Patients were followed up in line with standard clinical care for their injuries. They were asked to repeat the GHQ at two- and six-month follow-up.

Statistical analysis was undertaken on the Statistical Package for Social Sciences (SPSS v 16.0, SPSS Inc, Chicago, Ill, USA). Differences between GHQ caseness rates at different follow-up times were analysed using the Wilcoxon signed-ranks test. Normality of biomechanical data was tested using the Shapiro-Wilks test. Relationships between biochemical markers, categorical demographic data, and GHQ caseness were explored with the independent samples *t*-test, and those between the individual biochemical markers and GHQ scores with Pearson correlation testing. Logistic regression analysis was used to assess the predictive value of the biochemical markers on GHQ caseness.

## 3. RESULTS

During the course of the study, 84 patients were successfully recruited to the metabolic markers group. Their mean age was 36 years (range 16–68 years), and 75% were men. Fifty percent were married or cohabiting, while 45% were single, and 5% separated from partners. Alcohol consumption was greater than 20 units per week in 14 (17%), while 35% were smokers. Only one patient admitted the use of controlled substances, and eight had previous psychiatric services contact.

The mechanism of injury is summarised in Table 1. The patients had sustained a variety of musculoskeletal injuries, predominantly fractures, and 28 (35.5%) had multiple injuries. The modal ISS was nine (range 4–25), modal NISS was also nine (range 4–41). The mode RTS was 7.84 (range 6.38–7.84), and the modal TRISS survival probability was 99.40% (range 93.30–99.80%). Injuries were managed surgically in 89% of cases. Follow-up for the group was 68 (81%) at two months and 62 (74%) at six months.

The mean GHQ score at initial presentation was 1.31 (out of 28), rising at two months to 6.10, falling by six months to 3.39 (differences between follow up and baseline levels *P* < .001, Wilcoxon signed-ranks test). The initial caseness level was 11%, rising to 39% at two months, and falling back partially to 18% by six months (differences between follow-up and baseline *P* < .02, Wilcoxon signed-ranks test).

The mean standard deviation and the range for each of the biological markers are shown in Table 2. With the exception of GABA, each has a wide range. The measured quantity of each of the biological variables was normally distributed (Shapiro-Wilks test, *P* < .01). There were no correlations between levels of biological markers and demographic data including age (by age bands), sex, and type of injury (independent samples *t*-test, *P* > .05) and ISS score, NISS score, TRISS, and RTS (Pearson's correlation, *P* > .05). There were no correlations between GABA level and the levels of other biomarkers under investigation (IL-6, sIL6r, TNF-*α*, and CRP) (Pearson's correlation, *P* > .05).

 The levels of GABA, IL-6, sIL6r, TNF-*α*, and CRP did not correlate with GHQ total score at initial assessment and two- or six-month follow up (Pearson's correlation, *P* > .05). Similarly, the mean levels were not significantly different between cases and noncases (GHQ cut-off score five and above) (independent samples *t*-test, *P* > .05). Logistic regression analysis revealed that none of the biochemical markers had any predictive value for GHQ caseness (*P* > .10).

## 4. DISCUSSION

The relationship between markers of HPAA function after musculoskeletal injury and the risk of developing posttraumatic psychological problems is complex, and we have not been able to identify a simple predictive test from our data.

Posttraumatic psychopathology is an important complication of musculoskeletal trauma of the sort that presents every day to orthopaedic trauma surgeons. It is associated with a significant delay in functional recovery, and it is clear that genuinely holistic care of the injured patient should include the psychological state. Psychological interventions for PTP, including PTSD, are established, but are often limited in resource, and so should be targeted at those most in need.

We have previously reported on the relationship between psychological and physiological state after trauma [4], but have focused on incident-specific data in the prediction of at-risk status in our patients [9]. The recent suggestion that GABA may be useful in PTSD prediction in the short term [11] prompted our examination of GABA and proinflammatory markers of the metabolic response to trauma and their relationship to subsequent development of PTP.

Cortisol or ACTH assays are the most direct measures of HPA function and have been used to predict PTSD symnptomatology [33], but require complex methodology, particularly in specific timing of testing due to the effects of complex circadian rhythmns. Function of HPA and proinflammatory markers of trauma are enmeshed and complex, but it has been suggested that proinflammatory cytokine levels can provide a physiological correlation with HPA function [17]. It is not possible to identify a single marker of the inflammatory response to trauma. Each of the three cytokines (IL-6, sIL-6r, and TNF-*α*) that we have tested as well as CRP have been used in various studies of the response to physical trauma, including surgery [15, 34–48].

A focus upon PTSD as the sole psychiatric consequence of trauma is, in our view, too narrow. There is extensive symptom overlap in posttraumatic conditions, and while some patients will exhibit frank PTSD, others may not reach the diagnostic threshold while still suffering significant distress and dysfunction in their everyday life. For this reason, we have preferred to consider posttraumatic psychopathology (PTP) as representing the full spectrum of pathological reactions to injury, including associated depression or anxiety. This has led us to use GHQ as a screening tool for general psychiatric symptomatology, rather than focussing on a single diagnosis; we believe that this approach more truly reflects, in a practical way, the experience of our patients rather than in a research-driven enquiry.

Our data suggest no useful prediction of subsequent PTP by the assessment of HPA axis function, as measured by GABA, CRP, and proinflammatory cytokines. We have previously demonstrated the importance of the patient's perception of the incident, and would suggest that it more strongly predicts PTP than a snapshot of physiological markers [9].

## Figures and Tables

**Table 1:** Mechanism of injury (RTA = road traffic accident).

Mechanism		No. patients (%)
Fall	Total	29 (36)
	*Low energy*	*18 (22)*
	*From height*	*11 (14)*
Work including machinery		8 (10)
RTA	Total	35 (41)
	*Driver*	*12 (14)*
	*Passenger*	*7 (8)*
	*Motorcycle*	*11 (13)*
	*Cyclist*	*2 (2)*
	*Pedestrian*	*3 (4)*
Sport		11 (14)
Assault		1 (1)

**Table 2:** Biological markers levels.

Metabolic markers	*Initial* (*n* = 84)	
	Mean (SD)	Range
GABA (nmoL·L^−1^)	0.55 (0.243)	0.28–2.01
CRP (mg·L^−1^)	44 (49.81)	0–251.20
IL-6 (pg·mL^−1^)	83.75 (386.22)	0–3393.08
sIL-6r (pg·mL^−1^)	36361 (8418)	14587–66958
TNF-*α* (pg·mL^−1^)	27.37 (95.28)	0–754.01
